# Global Distribution, Dispersal Patterns, and Trend of Several Omicron Subvariants of SARS-CoV-2 across the Globe

**DOI:** 10.3390/tropicalmed7110373

**Published:** 2022-11-12

**Authors:** Ioannis Kopsidas, Sofia Karagiannidou, Evangelia Georgia Kostaki, Dimitra Kousi, Eirini Douka, Petros P. Sfikakis, Serafeim Moustakidis, Christos Kokkotis, Dimitrios Tsaopoulos, Ioulia Tseti, Theoklis Zaoutis, Dimitrios Paraskevis

**Affiliations:** 1 Center for Clinical Epidemiology and Outcomes Research (CLEO), 15451 Athens, Greece; 2 National Public Health Organisation (NPHO), 15123 Athens, Greece; 3 Department of Hygiene Epidemiology and Medical Statistics, Medical School, National and Kapodistrian University of Athens, 11527 Athens, Greece; 4 First Department of Propaedeutic and Internal Medicine, Medical School, National and Kapodistrian University of Athens, 11527 Athens, Greece; 5 AIDEAS OÜ, Narva mnt 5, 10117 Tallinn, Estonia; 6 Department of Physical Education and Sport Science, Democritus University of Thrace, 69100 Komotini, Greece; 7 Center for Research and Technology Hellas, Institute for Bio-Economy & Agri-Technology, 38333 Volos, Greece; 8 Uni-Pharma S.A., 14564 Kifissia, Greece

**Keywords:** SARS-CoV-2, coronavirus, Omicron, VOC, epidemiology, distribution, emergence, variants, subvariants

## Abstract

**Simple Summary:**

SARS-CoV-2 is characterized by extensive genetic divergence. The emergence of the variant of concern (VOC) Omicron, due to its characteristics, has caused an increased number of infections globally versus the previous VOCs. The omicron variant has been further divided into subvariants, with the most widely spread to be BA.1*, BA.2*, BA.3*, BA.4*, and BA.5*. Our aim was to analyze the global prevalence and the dispersal patterns of the different subvariants. Data about the proportions of the different variants over time and by geographic region were extracted from the CoV-Spectrum platform searching for BA.1*, BA.2*, BA.3*, BA.4*, and BA.5* variants. We showed that omicron subvariants spread at different time periods due to their biological properties and the characteristics of the pandemic. BA.5*, which caused the most recent pandemic wave globally, has dominated by the middle of August in Europe and elsewhere. Our study provides evidence that the ability of subvariants to dominate depends on their characteristics named transmissibility and immune evasion, given the characteristics of the SARS-CoV-2 immunity in the populations.

**Abstract:**

Our study aims to describe the global distribution and dispersal patterns of the SARS-CoV-2 Omicron subvariants. Genomic surveillance data were extracted from the CoV-Spectrum platform, searching for BA.1*, BA.2*, BA.3*, BA.4*, and BA.5* variants by geographic region. BA.1* increased in November 2021 in South Africa, with a similar increase across all continents in early December 2021. BA.1* did not reach 100% dominance in all continents. The spread of BA.2*, first described in South Africa, differed greatly by geographic region, in contrast to BA.1*, which followed a similar global expansion, firstly occurring in Asia and subsequently in Africa, Europe, Oceania, and North and South America. BA.4* and BA.5* followed a different pattern, where BA.4* reached high proportions (maximum 60%) only in Africa. BA.5* is currently, by Mid-August 2022, the dominant strain, reaching almost 100% across Europe, which is the first continent aside from Africa to show increasing proportions, and Asia, the Americas, and Oceania are following. The emergence of new variants depends mostly on their selective advantage, translated as enhanced transmissibility and ability to invade people with existing immunity. Describing these patterns is useful for a better understanding of the epidemiology of the VOCs’ transmission and for generating hypotheses about the future of emerging variants.

## 1. Introduction

The COVID-19 pandemic has led people and healthcare systems to an unprecedented situation globally. Since the beginning of the pandemic, several SARS-CoV-2 variants named after variants of concern (VOCs) have emerged, some of them highly transmissible (Alpha, Delta, Omicron), others showing partial immune escape (Beta, Omicron) or greater severity (Alpha, Delta) [1,2,3,4]. The Omicron variant was first detected in early November 2021 in Botswana, South Africa, and on the 26th of November 2021, was officially classified as a VOC by the World Health Organization (WHO) (https://www.who.int/news/item/26-11-2021-classification-of-omicron-(B.1.1.529)-sars-cov-2-variant-of-concern, accessed on 26 July 2022). After the initial detection in Africa, the Omicron variant spread rapidly around the globe and replaced the Delta variant.

The Omicron variant has shown a large number (more than 30) of mutations in the spike protein compared to the previous VOCs, a fact that induced scientific concerns with regard to possible increased transmissibility and immune escape: increased contagious window, variant shedding, environmental stability, and binding to host ACE2 receptors have all been reported as characteristics of the Omicron variant [5,6,7,8,9]. As for the sublineages BA.2, BA.4, and BA.5, which are currently the dominant subvariants, neutralization experiments have revealed that immunity induced by COVID-19 vaccines is less effective compared to previous VOCs [10,11,12,13]. Several countries have recommended a booster dose of COVID-19 vaccination in response to the emergence of the Omicron variant, still enlarging the immunization gap between high-income countries and countries in Africa, a fact that probably further favors the emergence of new variants [1].

Due to the high number of SARS-CoV-2 evolving variants, genomic surveillance is necessary to rapidly provide a real-time picture of the SARS-CoV-2 evolution globally, detecting newly appearing viral characteristics that could impact clinical outcomes [1]. The aim of this study was to describe the global distribution, dispersal patterns, and trend of the several Omicron subvariants of SARS-CoV-2 across the globe and European countries, based on data collected as part of genomic surveillance.

## 2. Materials and Methods

We extracted data from the CoV-Spectrum platform, which is designed to provide up-to-date genome data to help monitor variants [14]. The genomic data provided came from GISAID and unreleased/not yet released sequences from the Swiss SARS-CoV-2 Sequencing Consortium (S3C), complemented by metadata provided by the Federal Office of Public Health (FOPH) of Switzerland. We searched consecutively for BA.1*, BA.2*, BA.3*, BA.4*, and BA.5* and exported data as the proportion of all samples by geographic region (Africa, Asia, Europe, North America, South America, and Oceania). We combined the data sets and extracted data for the emergence of each variant by geographical area. Additionally, we looked at the distribution of BA.2* globally over time. We used the same strategy for the European countries. Finally, for the European countries, we calculated the time lag from the first day reported (first detection) to 50% dominance for the BA.1* and BA.2 subvariants*. Results were plotted as line charts and heatmaps.

## 3. Results

The different subvariants of omicron have emerged and dominated at different time points due to their different characteristics (i.e., infectivity, immune invasion).

### 3.1. BA.1* Subvariants Worldwide

**Figure 1:**
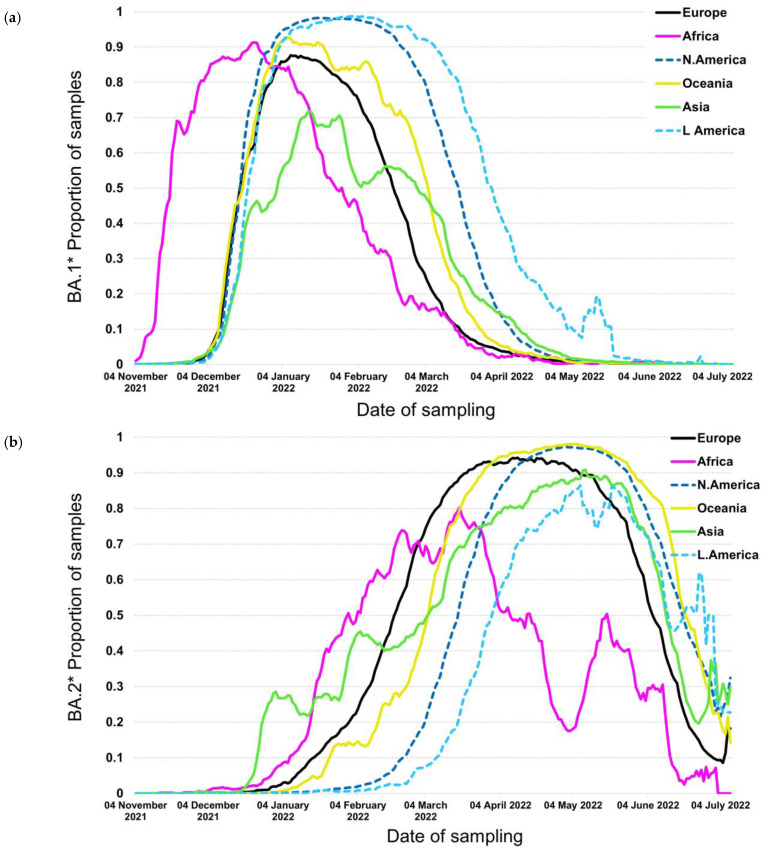
Proportion of samples over time by continent for (**a**) BA.1* and (**b**) BA.2*.

The BA.1*, including mainly the BA.1 and BA.1.1, were first detected in November 2021 and were the first subvariants to spread globally thereafter. Specifically, the proportions of BA.1* increased exponentially at the beginning of November 2021 in South Africa, with a similar increase to occur across all continents at the beginning of December 2021 (Figure 1a). The temporal characteristics of the BA.1* initial increase outside Africa coincided across different geographic areas, whereas it did not reach 100% dominance in all continents. The highest proportions were reached in America, followed by Oceania, Africa, and Europe, while in Asia, it reached only 70% during the initial omicron pandemic wave. The time period of the declining phase was not concurrent, as it was for the initial increase, with North and South America showing the most long-standing BA.1* pandemic wave.

### 3.2. BA.1* Subvariants in Europe

**Figure 2:**
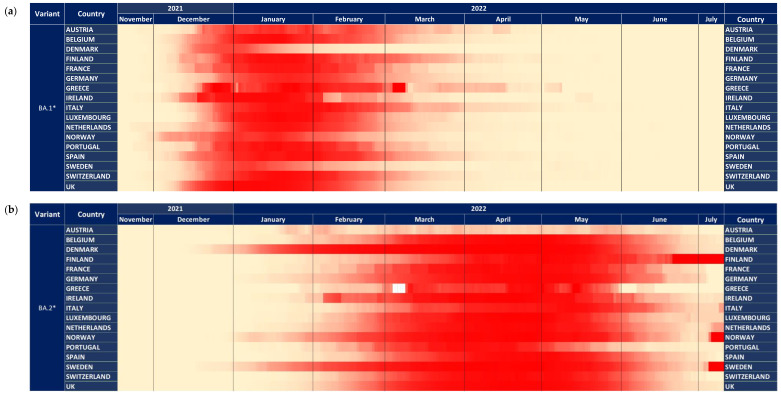
The dynamics of variants across 17 European countries shown with heatmaps representing the proportion of all samples. Heatmap shown with a maximum of 100% (**a**) BA.1* variants (**b**) BA.2* variants.

**Figure 3:**
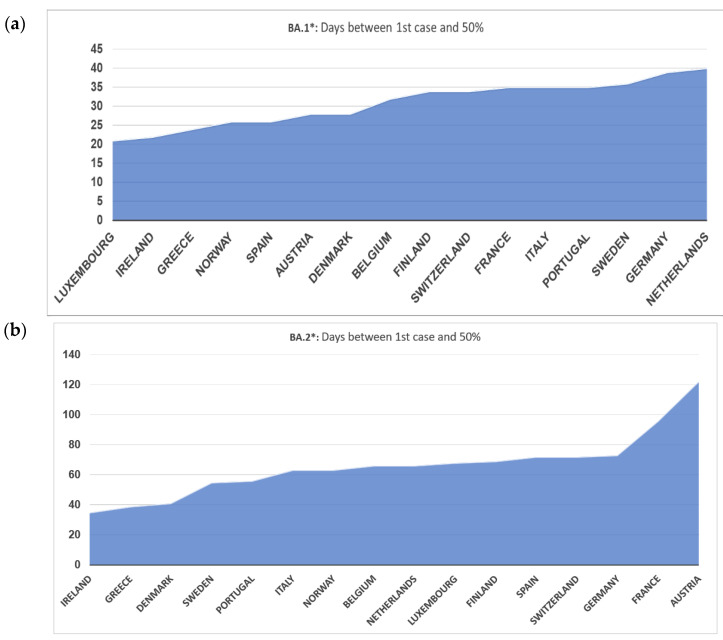
Days between first case and proportion of at least 50% of all samples (**a**) BA.1* variants (**b**) BA.2* variants.

Looking in more detail at the dynamics of BA.1* across Europe, this subvariant was first detected in Norway and subsequently in Ireland, Spain, Denmark, Finland, France, and Belgium (Figure 2a). Based on this figure, the earliest dispersal was detected in Norway, but high proportions were consistently found across Europe on the 20th of December 2021, with the Netherlands showing a later pattern of increase. The dominance of BA.1* followed a more diverse pattern, with Denmark and Sweden showing an early decreasing trend in the middle and early February, respectively (Figure 2a). The time lag between the first detection and the 50% proportion was between 20 and 40 days (Figure 3a). In Norway and Ireland, a subsequent decrease was detected that followed by most countries during March. The declining pattern of this subvariant did not follow a consistent time trend, as the early increase was observed during a period of 15–20 days.

### 3.3. BA.2* Subvariants Worldwide

The situation was very different with the BA.2* subvariants, which were first described in South Africa; however, their initial spread differed greatly with the geographic region. In contrast to BA.1*, which followed a similar global expansion, BA.2* initial spread first occurred in Asia and subsequently in Africa, Europe, Oceania, and North and South America (Figure 1b).

The time lag between the virus surge due to BA.2* in Africa and Europe was approximately 10–15 days, whereas the time lag between Europe and North America and Europe and South America was more pronounced and approximately equal to 1 and 1.5 months, respectively. The differences in the plateau proportions were obvious; in most areas, including North and South America, Oceania, Europe, and Asia, the maximum proportions were between 90% and almost 100%, but in Africa, although the plateau was reached earlier, the highest levels were lower than in other areas. The decline phase of BA.2* occurred within a shorter time period of approximately 14 days, except for Africa, which started much earlier. Among the rest areas, the decline started in Europe, followed by Asia, the Americas, and Oceania. The proportions of BA.1* were complementary to BA. 2 since the latter replaced the globally circulating BA.1*; therefore, the patterns of increase in BA.2* matched in a reverse manner the decline trends of BA.1*.

### 3.4. BA.2* Subvariants in Europe

The patterns of BA.2* followed a diverse time pattern that complemented the decreasing trends of BA.1* across Europe (Figure 2a,b). Specifically, BA.2* dominated first in countries with an early decrease in BA.1*, such as Denmark, Sweden, and Norway, followed by Ireland. For the rest of the countries, BA.2* showed a more similar pattern of increased proportions at the beginning of March (Figure 2b). The declining pattern was similar across Europe and occurred during May 2022. The time lag between first detection and 50% dominance was much larger for BA.2* compared to BA.1*, starting from 35 to approximately 70 days, with the exception of Austria and France, which was >100 days (Figure 3a,b).

### 3.5. Subvariants BA.4* and BA.5*

**Figure 4:**
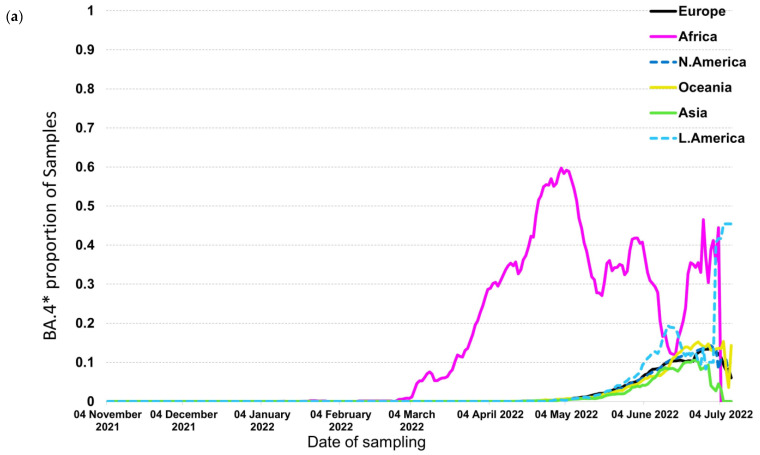

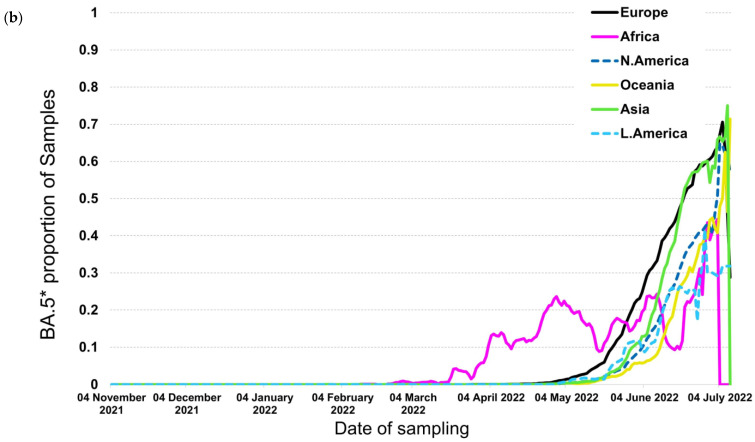
Proportion of samples over time by continent for (**a**) BA.4* and (**b**) BA.5*.

**Figure 5:**
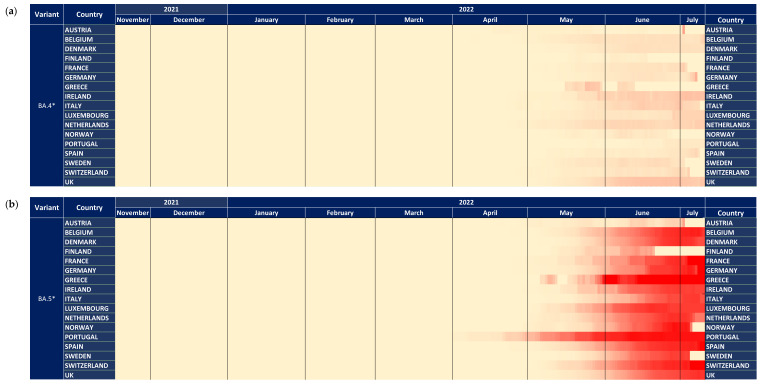
The dynamics of (**a**) BA.4*and (**b**) BA.5* variants across 17 European countries shown with heatmaps representing the proportion of all samples. All heatmaps shown with a maximum of 100%.

In contrast to the previous trends, BA.4* and BA.5*, which provide the currently circulating strains, followed a different pattern where BA.4* reached high proportions (maximum at ~60%) only in Africa (Figure 4a,b). The highest levels were approximately 10% in the other continents that lasted for a short period of fewer than 2 months. The patterns of BA.4* dispersal were very similar across the globe apart from Africa. The BA.5* proportions, on the other hand, are at high levels till now (date of database access: 12 July 2022), with Europe to be the first continent aside from Africa that showed increasing proportions and Asia, the Americas, and Oceania to be the following ones a small delay. A similar pattern was detected for BA.4* in Europe, where the increasing trend was low and occurred during May 2022 (Figure 5a). Regarding BA.5* in Europe, this subvariant showed a much earlier surge in Portugal that started in early April 2022 and was followed by other European countries with a time lag of approximately one month (Figure 5b). The earliest increase outside Portugal was found in Spain but with a small difference from the rest European countries. BA.5* provides the dominant strain that reached levels of almost 100% across Europe.

## 4. Discussion

The Omicron variant emerged in late 2021 (https://www.gisaid.org/, accessed on 26 July 2022) and was characterized as divergent due to the high number of mutations embedded in different genomic regions and especially in spike protein [9,15]. The Omicron viruses showed increased transmissibility and immune invasion compared to Delta, which was previously circulating across the globe, and the number of infections at the peak of the initial pandemic wave caused by this “new” variant was 5–10 times higher than the previous ones [15,16,17,18,19,20]. The Omicron dispersal started with subvariants BA.1*, which were gradually displaced by BA.2*, BA.4*, and BA.5* [4,21,22,23,24]. We did not include BA.3 in our analysis due to its low dispersal and thus limited importance for the pandemic.

The dynamics of BA.1* dispersal showed a coincidental increase in cases outside Africa as soon as the new subvariants were introduced in different geographic areas (Figure 1a). This was due to the large transmission advantage of the Omicron variant versus Delta that displaced the previous variant as soon as it entered all geographic areas [16,18,25]. The dominance of BA.1* was facilitated by the large population mobility between and within the country after the middle of December due to the Christmas and New Year Eve period. The rapid displacement of Delta by BA.1* that occurred both in areas of low, intermediate, or high vaccination coverage such as in Africa, Asia, or in Europe and the Americas, respectively, showed that besides high transmissibility, the new variant can invade more effectively individuals with existing immunity [15,18,26]. These characteristics have been reflected by the contemporaneous increase in Omicron across all continents outside Africa that occurred for the first time compared to previous VOCs, such as the Alpha, Beta, Gamma, or Delta [27]. Notably, a similar pattern was observed within Europe, with the time period of increase in BA.1* being almost identical among different countries. The proportions at the peak of BA.1* did not reach 100% globally due to the competition with the newly spreading subvariant of BA.2* that showed increased transmissibility compared to BA.1* (Figure 1a,b). As an exception, BA.1* reached high levels in the Americas (i.e., 100% over the course of the pandemic wave), where BA.2* emerged later and, therefore, there was no competition against BA.1*. The decreasing trend of BA.1* did not follow a stable temporal pattern due to the differences in the dynamics of BA.2* across the globe (Figure 1a,b). In areas where BA.2* had been introduced and started to spread, the proportion of BA.1* followed a declining trend. Interestingly, in the Americas, and particularly in Southern America, BA.2* was introduced later than the other areas, and probably this was due to more severe cross-border travel restrictions to enter the Americas compared to the rest of the world (Figure 1b) (https://www.cdc.gov/coronavirus/2019-ncov/travelers/proof-of-vaccination.html/, accessed on 26 July 2022).

The BA.2* dynamics followed a different pattern compared to BA.1*, and specifically, the time lag for the initial increase was incomparably higher than BA.1, with Asia and Europe showing the earliest out-of-Africa increase, followed by Oceania and the Americas. The time lag between Europe and North America was consistently observed also for alpha but not for Delta VOCs (https://www.GISAID.org/, accessed on 26 July 2022), for which the increase coincided in these areas. On the other hand, limited population mobility provides probably, the most plausible explanation for the delay in the introduction and spread of new variants in South America. The overall pattern of dispersal of BA.2* highlights that the advantage in transmissibility of these subvariants versus the previously circulating strains (BA.1*) was less pronounced than between BA.1* and Delta. This is highlighted by a non-contemporaneous increase in BA.2* versus BA.1*. BA.2* reached high levels at the peak of the pandemic wave, except for Africa, due to the co-circulation of the newly emergent and more fit BA.4* and BA.5* (Figure 4a,b). Large differences in the early dispersal of BA.2* have also been shown within Europe, where in some countries that were introduced earlier, it dominated BA.1* in February 2022 (Figure 2a). In all Scandinavian countries, BA.2* was introduced and spread earlier, and probably due to the limited population mobility from and to other non-Scandinavian countries, BA.2* did not spread widely during February (Figure 2b); however, this is a hypothesis that needs to be confirmed. On the contrary, within Scandinavian countries, BA.2* has spread earlier, and this provides another example of the significant role of population flow in the transmission of respiratory pathogens, as it has also been shown in the early transmission of COVID-19 in China [28,29,30,31].

The relative selective advantage of BA.5* versus BA.4* is probably higher compared to that of BA.4* versus BA.2*, as suggested by the dynamics of their transmission. Specifically, BA.4* reached low levels of dispersal outside Africa, and the dominance of BA.5* at the same time suggests that the latter has a significant selective advantage versus BA.4* and BA.2* (Figure 4a,b). Similarly, the increasing trend of BA.5* occurred approximately at the same time period for different continents, with Europe showing the earliest dispersal outside Africa. Like BA.2*, Portugal has shown the earliest transmission of BA.5* (Figure 5b).

Several sublineages (subvariants) have emerged, such as BA.4.6, BA.4.7, BA.5.9, and BQ.1 and BQ.1.1 that has been named recently by the European Centre for Disease Prevention and Control (ECDC) as a variant of Interest (VoI) characterized by increased immune escape ability (https://www.ecdc.europa.eu/en/publications-data/spread-sars-cov-2-omicron-variant-sub-lineage-bq1-eueea/, accessed on 26 July 2022).

Our study has a limitation related to different countries’ capacity to perform sequencing can significantly alter epidemiological surveillance data, thus making results difficult to interpret. To overcome this limitation, for a more detailed analysis, we selected European countries where sequencing bias is limited.

Overall, the emergence and dominance of new variants and subvariants depend mostly on their selective advantage, translated as enhanced transmissibility and ability to invade people with existing immunity. Their dynamics and dispersal patterns reflect the levels of their advantage compared to previously circulating strains, but also the global population’s mobility patterns, vaccination coverage, and public health measures. Our analysis provides additional data about the dispersal patterns of the Omicron subvariants that further support these findings. Genomic surveillance has been essential in detecting and assessing viral variants of interest and concern about the pandemic. Obtaining timely epidemiological about the newly emerging variants requires representative and targeted sampling of an adequate number of viruses circulating across different geographic areas as recommended by global public health organizations (https://www.ecdc.europa.eu/en/publications-data/guidance-representative-and-targeted-genomic-sars-cov-2-monitoring/, accessed on 26 July 2022). Proper and timely analysis of epidemiological data and dissemination to stakeholders is needed to ensure control of future pandemic waves. Looking into and analyzing these patterns can be useful for a better understanding of the epidemiology of VOCs transmission and for generating hypotheses about the future potential of newly emerging variants.

## Data Availability

Publicly available data were downloaded from the CoV-Spectrum platform (https://cov-spectrum.org, accessed on 26 July 2022) enabled by data from GISAID (https://gisaid.org/, accessed on 26 July 2022).
